# Bioenergetic Dynamics of Heat Exchange in Japanese Quail Under Heat Stress with *Gracilaria birdiae* Supplementation

**DOI:** 10.3390/ani16162547

**Published:** 2026-08-14

**Authors:** Ricardo de Sousa Silva, Dermeval Araújo Furtado, Carlos Eduardo Alves Oliveira, Airton Gonçalves de Oliveira, Neila Lidiany Ribeiro, Tácila Rodrigues Arruda, José Pinheiro Lopes Neto, Matteo Barbari

**Affiliations:** 1 Academic Unit of Agricultural Engineering, Center for Technology and Natural Resources, Federal University of Campina Grande (UFCG), Campina Grande 58429-900, PB, Brazil; ricardo.s.silva@estudante.ufcg.edu.br (R.d.S.S.); dermeval.araujo@professor.ufcg.edu.br (D.A.F.); tacila.rodrigues@estudante.ufcg.edu.br (T.R.A.); jose.pinheiro@professor.ufcg.edu.br (J.P.L.N.); 2 Coordination of Agricultural Engineering, Chapadinha Science Center, Federal University of Maranhão, Chapadinha 58397-000, MA, Brazil; airton.oliveira@ufma.br; 3 Department of Animal Science, Center for Agricultural Sciences, Federal University of Paraíba, Areia 65500-000, PB, Brazil; nlr@academico.ufpb.br; 4 Department of Agriculture, Food, Environment and Forestry, University of Firenze, 50145 Firenze, Italy

**Keywords:** biophysical modeling, homeothermy, poultry physiology, respiratory evaporative heat loss, sensible heat dissipation

## Abstract

The intensification of poultry production, associated with climate change, has increased the occurrence of heat stress, compromising animal welfare and productive efficiency. Despite recent advances, studies quantifying heat exchange in quail under different environmental and dietary conditions from an integrated bioenergetic perspective remain scarce, particularly regarding the shift between sensible and latent heat dissipation mechanisms. In this context, this study aimed to quantify and model sensible and latent heat exchange, together with associated physiological responses, in Japanese quail (*Coturnix coturnix japonica*) subjected to different air temperatures and dietary inclusion levels of the macroalga *Gracilaria birdiae*. A total of 864 quail were distributed in a completely randomized design, arranged in a 4 × 3 factorial design with four macroalgae inclusion levels (0.00, 3.00, 6.00, and 9.00%) and three air temperature levels (25.00, 29.00, and 33.00 °C), and maintained in climate-controlled chambers. Heat exchange was estimated using biophysical models integrating convective, radiative, and evaporative heat fluxes. Increasing air temperature reduced sensible heat exchange and intensified latent heat losses (*p* < 0.0001). During the growing phase, approximately 73.18% of sensible heat exchange was dissipated through radiation. In the laying phase, reductions of up to 59.96% in sensible heat exchange were observed, along with increases exceeding 50.00% in latent heat losses and reductions of up to 26.00% in total heat exchange. Increasing air temperature promoted higher respiratory rate (*p* < 0.0001), whereas surface and cloacal temperatures remained within the physiological range required to maintain homeothermy. Dietary inclusion of up to 9.00% *G. birdiae* exerted only limited effects on the quantified heat exchange pathways and did not impair physiological thermoregulation under the experimental conditions evaluated. No significant interaction between air temperature and dietary supplementation was observed for the heat exchange variables (*p* > 0.05). These findings show that heat stress was the primary determinant of bioenergetic heat exchange, whereas dietary supplementation with *G. birdiae* exerted only limited effects.

## Simple Summary

Climate change has increased the frequency of periods of intense heat, making it more difficult to maintain the welfare and productivity of poultry. Japanese quail are particularly sensitive to high temperatures and must continuously adjust how they release excess body heat to keep their body temperature within a safe range. In this study, we evaluated how different air temperatures and the inclusion of the marine macroalga *Gracilaria birdiae* in the diet influenced these adaptive responses. The results showed that, as air temperature increased, the quails relied more on heat loss through respiration, while the mechanisms normally used under milder conditions became less effective. Despite these adjustments, the quail maintained their body temperature within the normal physiological range. Dietary inclusion of up to 9.00% *G. birdiae* did not impair the quails’ ability to regulate body temperature. These findings improve our understanding of how Japanese quail respond to heat stress and provide information that may support the development of nutritional and management strategies to improve animal welfare and poultry production under climate change.

## 1. Introduction

Japanese quail (*Coturnix coturnix japonica*) has become one of the most important avian species for commercial egg and meat production because of its early sexual maturity, rapid growth, high feed efficiency, and low space requirements [1,2]. In addition to its economic importance, the species has become an established experimental model for studies on animal nutrition, physiology, reproduction, and production systems owing to its well-characterized biological responses and suitability for controlled experimental conditions [3,4]. The continuous expansion of Japanese quail production has increased the need to better understand the physiological mechanisms that determine animal performance and welfare under different environmental conditions, particularly those associated with thermal stress [1,3].

Climate change has increased the frequency, intensity, and duration of heat waves [5,6,7], exposing Japanese quail production systems to progressively greater thermal challenges. Owing to their high metabolic rate and limited capacity for heat dissipation under hot environmental conditions, Japanese quail are particularly susceptible to heat stress, which compromises productive performance, welfare, and physiological homeostasis [8]. Elevated ambient temperatures have been associated with reduced feed intake, impaired growth and egg production, increased respiratory effort, oxidative stress, and disturbances in acid–base balance, demonstrating the marked physiological sensitivity of this species to thermal stress [8,9]. These responses highlight the importance of understanding the physiological mechanisms underlying thermal adaptation in Japanese quail [10].

Maintaining homeothermy depends on a continuous balance between metabolic heat production and heat dissipation to the surrounding environment. Under thermoneutral conditions, this balance is achieved primarily through sensible heat exchange by radiation, convection, and conduction, whose efficiency depends on the thermal gradient between the body surface and the surrounding air [10,11,12]. As ambient temperature increases, this gradient progressively decreases, reducing the effectiveness of sensible heat dissipation. Because birds lack sweat glands and their plumage limits cutaneous evaporative cooling, respiratory evaporation progressively becomes the primary avenue for heat loss under hot environmental conditions [10,11,13]. In Japanese quail, air temperatures above 29.00 °C progressively reduce the contribution of sensible heat exchange, increasing the reliance on latent heat dissipation to maintain homeothermy [10,14]. Although this physiological adjustment is essential for survival, its prolonged activation increases energy expenditure and water demand and may compromise productive performance under sustained heat stress [11,15].

The efficiency of thermoregulatory mechanisms depends on whether birds remain within their thermoneutral zone, in which body temperature is maintained with minimal physiological and metabolic effort [16,17]. Experimental studies with adult Japanese quail generally consider ambient temperatures between approximately 22.00 and 26.00 °C as thermoneutral conditions, whereas temperatures above this range progressively increase the physiological demand for active thermoregulation and evaporative heat dissipation [11,17,18]. Consequently, prolonged exposure to temperatures exceeding the upper limit of thermoneutrality increases the energetic cost of thermoregulation [10,12].

Although environmental control systems are widely adopted in commercial poultry production, they do not completely eliminate the occurrence of heat stress, particularly during prolonged heat waves, periods of high thermal load, or situations involving limitations in ventilation efficiency, cooling capacity, or energy supply [14,19]. Consequently, Japanese quail may still experience environmental conditions that challenge their thermoregulatory capacity and compromise productive performance and welfare [8,9]. Controlled-environment experiments are essential because they isolate physiological and bioenergetic responses to specific thermal conditions, providing mechanistic evidence that cannot be obtained reliably under commercial production systems [10,11]. This experimental approach also provides a robust framework for evaluating complementary strategies to improve the physiological resilience of Japanese quail exposed to heat stress.

Functional nutritional strategies have been investigated as complementary approaches to improve the physiological resilience of Japanese quail exposed to heat stress by supporting homeostasis, intestinal integrity, and oxidative balance [15,20,21,22]. Among these strategies, marine macroalgae have attracted increasing attention as sustainable sources of bioactive compounds with antioxidant and anti-inflammatory properties [2,15,23]. In particular, *Gracilaria birdiae*, a marine macroalga native to the Brazilian coast, has demonstrated beneficial effects on the productive performance and physiological responses of Japanese quail reared under hot environmental conditions [15,22]. However, whether this supplementation influences the bioenergetic dynamics of sensible and latent heat exchange during heat stress remains unknown.

Despite recent advances in the use of marine macroalgae and other functional feed additives to mitigate the effects of heat stress in Japanese quail, available studies have primarily focused on productive, physiological, and antioxidant responses, whereas quantitative assessments of the bioenergetic dynamics of sensible and latent heat exchange remain scarce [22,24]. To the best of our knowledge, no studies have evaluated these heat exchange mechanisms in Japanese quail supplemented with *Gracilaria birdiae* under controlled thermal conditions, preventing a mechanistic understanding of the interaction between functional nutrition and thermoregulation. Therefore, we hypothesized that dietary supplementation with *G. birdiae* might influence the bioenergetic dynamics of heat exchange during heat stress, potentially influencing the relative contribution of sensible and latent heat dissipation pathways during heat stress.

Based on this hypothesis, this study aimed to quantify the dynamics of sensible and latent heat exchange and the associated physiological responses in Japanese quail (*Coturnix coturnix japonica*) fed increasing dietary levels of the marine macroalga *Gracilaria birdiae* and exposed to different controlled air temperatures using an integrated bioenergetic modeling approach.

## 2. Materials and Methods

### 2.1. Study Site, Ethical Approval, and Climate Chambers

The study was conducted at the Laboratory of Rural Buildings and Environment (LaCRA), Academic Unit of Agricultural Engineering, Federal University of Campina Grande (UAEA/UFCG), located in Campina Grande, Paraíba, Brazil (07°12′52″ S; 35°54′26″ W; 528 m above sea level). All experimental procedures involving animals were approved by the Animal Ethics Committee of the Federal University of Campina Grande (CEUA/UFCG; Protocol No. 11/2024; approved on 28 March 2024) and were conducted in accordance with the institutional guidelines for the care and use of animals in research. The study was reported in compliance with the ARRIVE 2.0 guidelines. No prospective study protocol specifying the research objectives, experimental design, or statistical analysis plan was prepared before the study was initiated.

**Figure 1:**
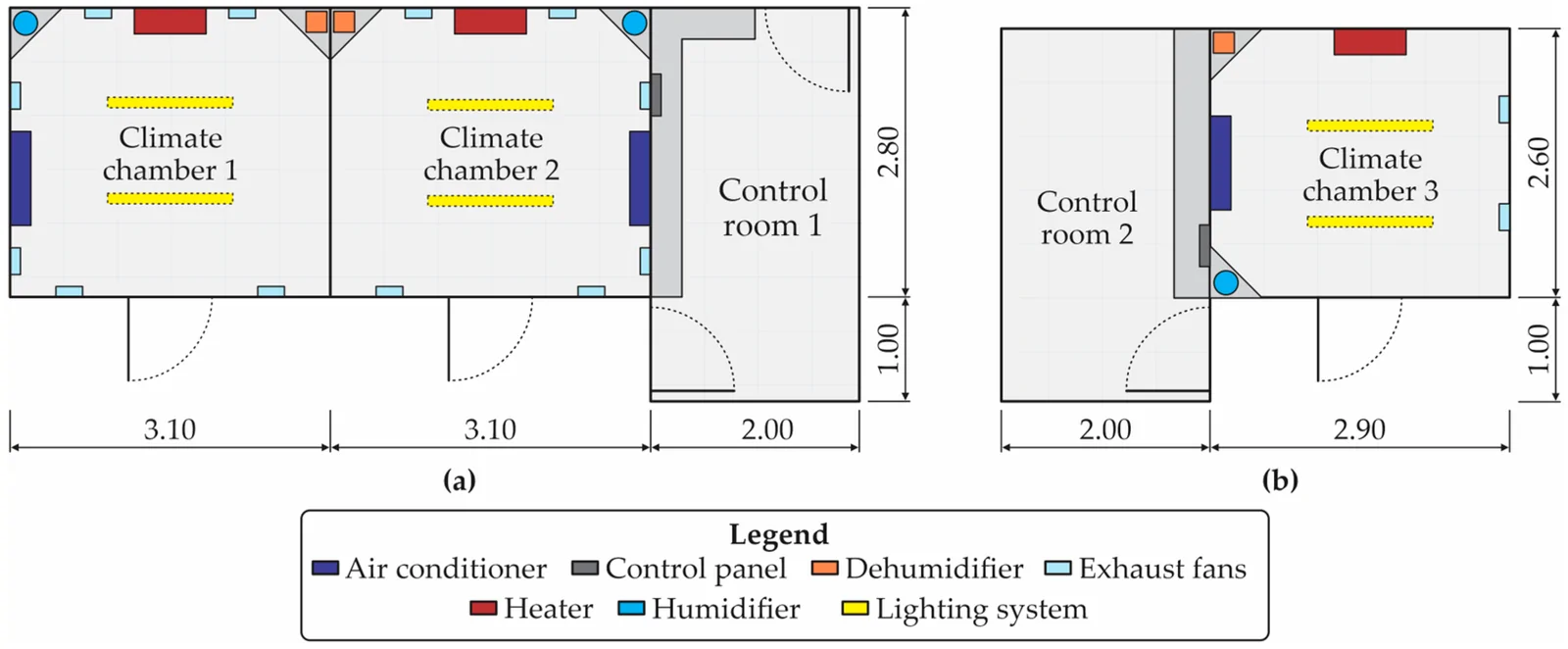
Layout of the experimental infrastructure used in the study, showing the climate chambers and their respective monitoring rooms, including (**a**) climate chambers 1 and 2 and (**b**) climate chamber 3. Dimensions are expressed in meters.

Experimental procedures were conducted under controlled environmental conditions using three climate chambers available at LaCRA/UAEA/UFCG (Figure 1). Climate chambers 1 and 2 had internal dimensions of 3.10 × 2.80 × 2.50 m (length × width × height), whereas climate chamber 3 measured 2.90 × 2.60 × 2.50 m. All chambers were constructed with galvanized steel panels coated with an anticorrosive layer and insulated with expanded polystyrene (EPS), providing effective thermal insulation from the external environment. This experimental setup enabled the simultaneous evaluation of independent thermal treatments under control and reproducible environmental conditions. The experimental infrastructure has been used in previous studies on thermal physiology, animal environment, and biophysical modeling under controlled environmental conditions, demonstrating operational stability and experimental reproducibility [10,25,26]. The microclimatic conditions were controlled by an automated system comprising air conditioners, electric heaters, humidifiers, dehumidifiers, and exhaust fans, allowing automatic regulation of air temperature and relative humidity within each experimental environment. The equipment was strategically arranged to promote a uniform distribution of microclimatic conditions throughout each climate chamber. Operational control was performed using MT-530 PLUS microprocessor-based electronic controllers (Full Gauge Controls, Canoas, RS, Brazil), featuring a control resolution of 0.10 °C, an NTC temperature sensor input, and independent outputs for the automatic operation of the cooling and heating systems. The controllers were integrated with the SITRAD^®^ software (version 1.8.25; Full Gauge Controls, Canoas, RS, Brazil) for continuous monitoring and recording of environmental conditions.

Figure 1 illustrates the spatial layout of the climate chambers, the monitoring room, and the arrangement of the main equipment used for environmental control within the experimental facilities. The procedures for microclimatic monitoring, the instrumentation employed, the environmental variables measured, and the biophysical models used to estimate heat exchange are described in Section 2.3.

### 2.2. Animals, Experimental Design, and Dietary Treatments

**Figure 2:**
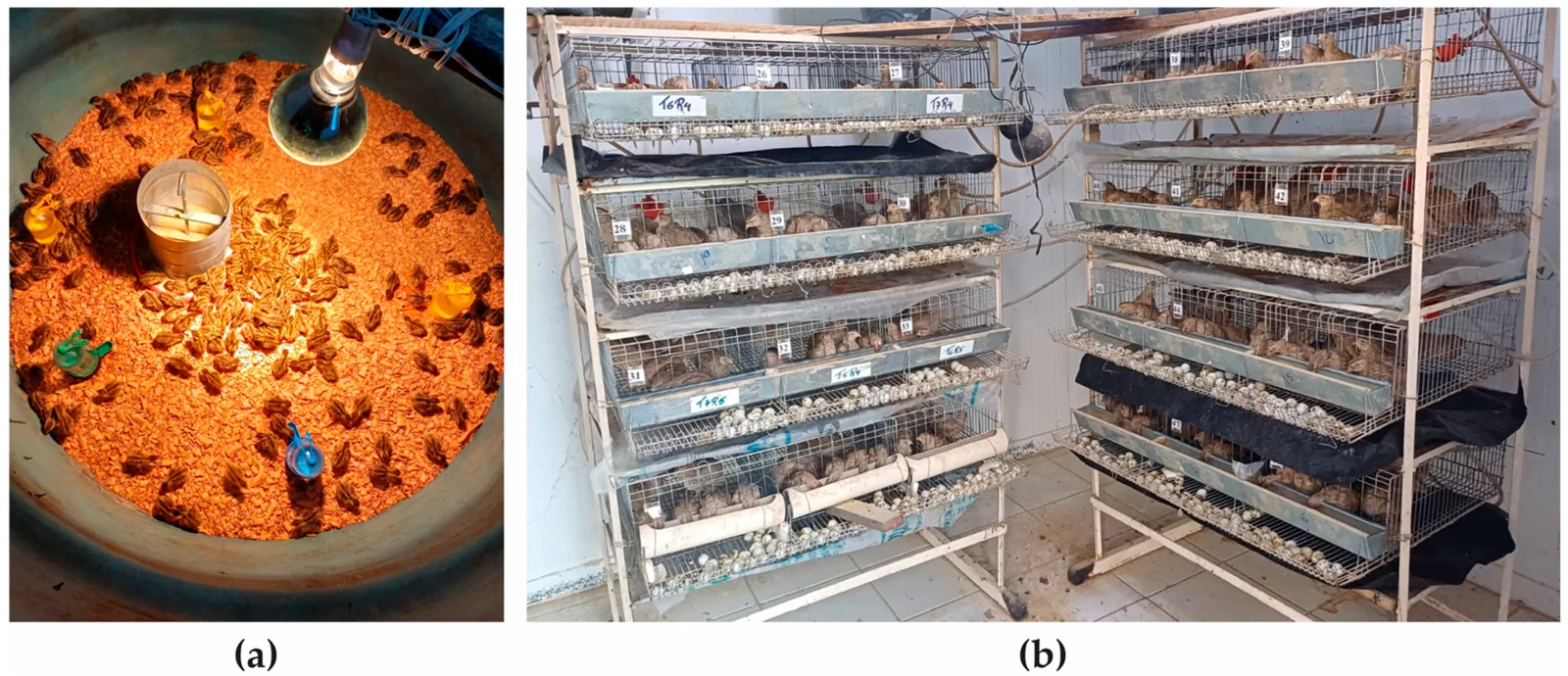
Experimental housing conditions adopted during the adaptation and thermal challenge phases: (**a**) brooding system used during the 14-day adaptation period; and (**b**) housing of Japanese quail inside controlled-environment climatic chambers during the experimental period.

A total of 864 one-day-old female Japanese quail (*Coturnix coturnix japonica*) with an initial body weight of 6.70 ± 0.50 g were obtained from a commercial breeding flock with certified genetic purity (Granja Fujikura, Suzano, SP, Brazil). During the first 14 days of age, the quail were maintained in the brooding phase, housed in brooding circles containing wood-shaving bedding, and provided with artificial heating using continuously operated 60 W incandescent lamps (Figure 2a). During this period, air temperature was maintained between 32.00 and 34.00 °C (±0.50 °C), while relative humidity ranged from 60.00 to 70.00% (±2.00%) to provide appropriate microclimatic conditions for early quail development. Feed and water were provided *ad libitum* throughout the adaptation period. The quail were clinically healthy throughout the adaptation period and received routine health management, including vaccination and deworming. At the end of the adaptation period, all birds were individually weighed and randomly allocated to the experimental units of the completely randomized factorial design. Random allocation was performed after body weight assessment to ensure a balanced initial distribution of body weight among treatments and to minimize potential allocation bias. At the end of the adaptation period, the quails had reached a mean body weight of 50.10 ± 0.23 g and were then transferred to the climate chambers for the beginning of the experimental period.

Following random allocation, the experimental units were established according to a completely randomized 4 × 3 factorial design, consisting of four dietary inclusion levels of the marine macroalga *G. birdiae* (0.00%, corresponding to the control diet; 3.00%; 6.00%; and 9.00%) and three controlled air temperature conditions (25.00 °C; 29.00 °C; and 33.00 °C). The selected dietary inclusion levels were established based on previous experimental studies evaluating *G. birdiae* supplementation in Japanese quail, ensuring experimental continuity and facilitating direct comparison with previous studies [15,26]. The selected air temperatures were established based on previous studies describing the thermoneutral zone and the progressive physiological responses of Japanese quail to increasing thermal challenge, representing conditions ranging from thermoneutrality to heat stress [17,18]. Each thermal condition was assigned to an independent climate chamber housing 288 quails. Owing to the available experimental infrastructure, one climate chamber was assigned to each thermal condition, whereas dietary treatments were randomly distributed among cage compartments within each chamber. Within each climate chamber, the experimental units assigned to the four dietary treatments were randomly distributed among cage compartments, each containing an independent group of birds that was maintained throughout the experimental period, in order to minimize possible positional effects within the housing system. Blinding was not implemented during animal allocation, experimental management, physiological measurements, or statistical analysis because dietary supplementation levels and controlled environmental conditions were readily identifiable throughout the study. The quails were housed in galvanized wire cage batteries (Figure 2b) arranged in four vertical tiers, with each cage divided into three experimental compartments (1.00 × 0.50 × 0.15 m). Each compartment represented one experimental unit, measured 0.33 × 0.50 × 0.15 m, provided a usable floor area of approximately 0.17 m^2^, and housed 12 quails, corresponding to a stocking density of approximately 72 birds m^−2^. The experiment comprised 72 experimental units distributed into 12 treatments, each with six replicates, totaling 864 quails.

The experimental period was conducted from 15 to 112 days of age. Following the 14-day adaptation period, quails remained under their assigned dietary and thermal treatments throughout the experimental period, during which environmental and physiological measurements were performed according to the predefined sampling schedule. During the growing phase, quails were maintained under a lighting program of 22 h light and 2 h darkness. At the onset of the laying phase, the lighting program was adjusted to 16 h light and 8 h darkness, consistent with the lighting regimen traditionally adopted for laying Japanese quail [27]. Feed and water were provided *ad libitum*. The experimental diets consisted of four dietary inclusion levels of *G. birdiae* (0.00, 3.00, 6.00, and 9.00%), corresponding to the nutritional treatments evaluated in this study. The ingredient composition and calculated nutritional composition of the experimental diets are listed in Table 1. All diets were formulated to meet the nutritional requirements of Japanese quail during the growing and laying phases while maintaining equivalent levels of metabolizable energy and nutrients across treatments [28]. No environmental enrichment was provided because the experimental objective required standardized housing conditions and strict control of environmental variables across treatments.

The marine macroalga *G. birdiae*, used as a functional ingredient in the experimental diets, was purchased in dehydrated form from a commercial supplier located on the coast of Santa Catarina State, Brazil. The dried material was subsequently ground to obtain a particle size suitable for homogeneous incorporation into the experimental diets. The chemical characterization of the raw material was performed prior to diet formulation at the Laboratory of Storage and Processing of Agricultural Products (LAPPA/UAEA/UFCG) and the Food Engineering Laboratory (LEA/UFCG). The analyses included the determination of mineral composition and the major bioactive compounds. The analyses were performed by specialized laboratories following their routine validated analytical procedures for food and feed characterization. All analytical determinations were performed in triplicate, and the results are expressed as the mean of three independent measurements. The mineral composition, major bioactive compounds, and proximate composition of the macroalga used in this study are listed in Table 2. Because crude fiber was not included among the analytical determinations performed for this batch, no values from the literature were incorporated owing to the well-documented variability in the chemical composition of marine macroalgae.

No predefined exclusion criteria were established for animals or experimental units before the study. All clinically healthy quails were eligible for inclusion in the experiment following the adaptation period. Throughout the experimental period, no animals or experimental units were excluded from the analyses. Consequently, all 864 quails distributed among the 72 experimental units remained in the experiment and were included in the statistical analysis.

### 2.3. Environmental Monitoring, Physiological Measurements, and Heat Exchange Modeling

The microclimatic conditions inside the climate chambers were monitored using an automated data acquisition system integrated with the environmental control system described in Section 2.1. Air temperature was measured using DS18B20 digital temperature sensors (Analog Devices^®^, Wilmington, MA, USA), with a measurement range of −55.00 to 125.00 °C and an accuracy of ±0.50 °C over the range of −10.00 to 85.00 °C. Relative humidity was monitored using DHT22 digital sensors (Aosong Electronics^®^, Guangzhou, China), with a measurement range of 0.00 to 100.00%, an accuracy of ±2.00%, and a resolution of 0.10%. The sensors were installed within the occupied zone of the quails at the geometric center of the cages to accurately represent the microclimatic conditions experienced by the animals. Both sensors were connected to an ESP32 microcontroller (Espressif Systems^®^, Shanghai, China) operating at 3.30 V and equipped with IEEE 802.11 b/g/n Wi-Fi and Bluetooth v4.2 BR/EDR and BLE connectivity for environmental data acquisition and processing. Air velocity was measured using an LM8000 digital thermo-hygro-anemometer-lux meter (Akso^®^, São Leopoldo, RS, Brazil), with a measurement range of 0.40 to 30.00 m s^−1^, a resolution of 0.10 m s^−1^, and an accuracy of ±3.00% of the reading or ±0.20 m s^−1^. Environmental variables were recorded at 30 min intervals throughout the experimental period and subsequently processed for use as input variables in the biophysical models employed to estimate heat exchange and interpret the thermoregulatory responses of the quails.

Physiological measurements and heat exchange modeling were performed during the growing (15 to 42 days of age) and laying (49 to 112 days of age) phases. Physiological variables were measured twice weekly (Tuesdays and Fridays) at 08 a.m. using four quails per experimental unit. Respiratory rate (RR, breaths min^−1^), body surface temperature (T_S_, °C), and cloacal temperature (T_C_, °C) were measured and subsequently used to interpret the thermoregulatory responses and model heat exchange [11,15,25]. The RR was determined by directly counting respiratory movements for 20 s, and the values were subsequently converted to breaths per minute. The T_S_ was measured using an infrared thermometer (Instrutherm^®^, model TI-870; Instrutherm Instrumentos de Medição Ltda., São Paulo, SP, Brazil; measurement range: −50.00 to 380.00 °C; resolution: 0.10 °C; accuracy: ±0.20 °C). Measurements were obtained from the head, back, ventral region beneath the wing, and shanks. The T_C_ was measured using a digital clinical thermometer (Incoterm^®^, model MC-245; Incoterm Indústria de Termômetros Ltda., Porto Alegre, RS, Brazil; measurement range: 32.00 to 42.00 °C; resolution: 0.10 °C; accuracy: ±0.10 °C), inserted approximately 20 mm into the cloaca until the reading stabilized. All physiological measurements were performed by the same operator immediately after the quails were removed from the cages to minimize handling-induced physiological alterations. All handling procedures were performed by trained personnel following standardized protocols to minimize handling time and animal stress. These physiological measurements, together with the biophysical estimates of convective (E_Cv_), radiative (E_Ra_), total sensible (E_SH_), respiratory latent (E_La_), and total heat exchange (E_TH_) described below, comprised the outcome measures evaluated in this study.

The mean body surface temperature (T_S_, °C) was estimated by applying a weighted average to the temperatures measured at the different anatomical regions according to the relative contribution of each region to the effective body surface area, following the methodology proposed by Richards [29]. This procedure provides a physiologically representative estimate of the mean body surface temperature by minimizing the effect of thermal heterogeneity among body regions, thereby allowing its use as an input variable for the biophysical modeling of heat exchange [15,25]. Equation (1) describes the mathematical procedure used to calculate T_S_.

(1) $$T_{S}=0.12\times T_{wing}+0.03\times T_{head}+0.15\times T_{shank}+0.70\times T_{back}$$

where $T_{wing}$ is the wing surface temperature (°C); $T_{head}$ is the head surface temperature (°C); $T_{shank}$ is the shank surface temperature (°C); and $T_{back}$ is the back surface temperature (°C).

Sensible heat exchange between the quail and the surrounding environment was estimated using a biophysical model based on the energy balance approach proposed by Turnpenny et al. [30], in which the total sensible heat flux is defined as the sum of the convective and radiative components. Because the experiments were conducted in closed climate chambers, without direct solar radiation, and the galvanized wire floor and perches remained close to ambient temperature after thermal stabilization, the limited contact area between the quails and the supporting structures, together with the small thermal gradient at the contact surfaces, resulted in negligible conductive heat exchange relative to convective, radiative, and evaporative heat transfer, consistent with previous bioenergetic studies under controlled environmental conditions [10,31]. Accordingly, sensible heat exchange (E_SH_, in W m^−2^) was calculated using Equation (2).

(2) $$E_{SH}=E_{Cv}+E_{Ra}$$

where $E_{Cv}$ is the convective heat exchange (W m^−2^) and $E_{Ra}$ is the net longwave radiative heat exchange (W m^−2^).

The convective component of sensible heat exchange was estimated according to the biophysical model proposed by McArthur [31], which considers heat transfer between the bird’s body surface and the surrounding air as a function of the temperature gradient and the boundary layer resistance to airflow. In this model, convective heat exchange (E_Cv_, in W m^−2^) was calculated using Equation (3):

(3) $$E_{Cv}=\frac{\rho \times c_{p}\times (T_{S}-T_{A})}{r_{Cv}}$$

where ρ is the air density (kg m^−3^); $c_{p}$ is the specific heat capacity of air (J kg^−1^ K^−1^); $T_{S}$ is the mean body surface temperature (K); $T_{A}$ is the air temperature (K); and $r_{Cv}$ is the boundary layer resistance to convective heat transfer (m^2^ K W^−1^).

The boundary layer resistance to convective heat transfer (r_Cv_, in m^2^ K W^−1^) was estimated by considering the thermophysical properties of air, the characteristic dimension of the bird, and the airflow conditions around the body surface. This parameter was calculated using Equation (4):

(4) $$r_{Cv}=\frac{\rho \times c_{p}\times d_{B}}{k\times Nu}$$

where ρ is the air density (kg m^−3^); $c_{p}$ is the specific heat capacity of air (J kg^−1^ K^−1^); $d_{B}$ is the mean body diameter (m); k is the thermal conductivity of air (W m^−1^ K^−1^); and Nu is the Nusselt number (dimensionless).

The mean body diameter of the quails (d_B_, in m), used as the characteristic dimension for estimating convective heat transfer between the bird’s body surface and the surrounding air, was determined from the mean body weight at each experimental age using the allometric relationship proposed by Mitchell [32], as described in Equation (5):

(5) $$d_{B}=\frac{0.131\times m^{0.33}}{100}$$

where m is the mean body weight (g).

Assuming a simplified geometric representation of the bird’s body as a sphere and forced convection around the animal, the Nusselt number (Nu, dimensionless) was estimated using the empirical correlation proposed by Whitaker [33], which is widely used to describe convective heat transfer from spheres subjected to external flow, as given by Equation (6).

(6) $$Nu=2+(0.40\times Re^{\frac{1}{2}}+0.06\times Re^{2/3})\times {Pr}^{0.4}$$

where Re is the Reynolds number (dimensionless) and Pr is the Prandtl number (dimensionless).

The Reynolds number (Re, dimensionless) was calculated to characterize the airflow regime around the quails’ body surface, as given by Equation (7):

(7) $$Re=\frac{v_{air}\times d_{B}}{\nu }$$

where $v_{air}$ is the air velocity (m s^−1^); $d_{B}$ is the mean body diameter (m); and ν is the kinematic viscosity of air (m^2^ s^−1^).

The Prandtl number (Pr, dimensionless) was calculated using Equation (8):

(8) $$Pr=\frac{\rho \times c_{p}\times \nu }{k}$$

where ρ is the air density (kg m^−3^); $c_{p}$ is the specific heat capacity of air (J kg^−1^ K^−1^); ν is the kinematic viscosity of air (m^2^ s^−1^); and k is the thermal conductivity of air (W m^−1^ K^−1^).

The values of Re and Pr were subsequently used to determine the Nusselt number (Equation (6)), which was used to estimate the boundary layer resistance to convective heat transfer.

Longwave radiative heat exchange ($E_{Ra}$, in W m^−2^) was also estimated according to the model proposed by McArthur, considering only the longwave thermal radiation component, since the experiment was conducted in climate chambers without exposure to direct solar radiation (Equation (9)) [30,31].

(9) $$E_{Ra}=\frac{\rho \times c_{p}\times (T_{S}-T_{MR})}{r_{Ra}}$$

where ρ is the air density (kg m^−3^); $c_{p}$ is the specific heat capacity of air (J kg^−1^ K^−1^); $T_{S}$ is the mean body surface temperature (K); $T_{MR}$ is the mean radiant temperature (K); and $r_{Ra}$ is the boundary layer resistance to radiative heat transfer (m^2^ K W^−1^).

The boundary layer resistance to radiative heat transfer was calculated using Equation (10).

(10) $$r_{Ra}=\rho \times c_{p}\times {\left(4\times \varepsilon_{S}\times \sigma \times {\bar{T_{M}}}^{3}\right)}^{-1}$$

where ρ is the air density (kg m^−3^); $c_{p}$ is the specific heat capacity of air (J kg^−1^ K^−1^); $\varepsilon_{S}$ is the surface emissivity of the feathers, assumed to be 0.94; σ is the Stefan–Boltzmann constant (5.67051 × 10^−8^ W m^−2^ K^−4^); and $\bar{T_{M}}$ is the mean of $T_{S}$ and $T_{MR}$ (K).

The mean radiant temperature (T_MR_, in K) was estimated from the black globe temperature using the relationship proposed by Silva [34], as given by Equation (11).

(11) $$T_{MR}={\left[\frac{1.053\times h_{Cv}}{\sigma }\times \left(T_{BG}-T_{A}\right)+T_{BG}^{4}\right]}^{1/4}$$

where $T_{BG}$ is the black globe temperature (K); $T_{A}$ is the air temperature (K); and $h_{Cv}$ is the globe convective heat transfer coefficient (W m^−2^ K^−1^).

The convective heat transfer coefficient of the black globe (h_Cv_, in W m^−2^ K^−1^) was calculated using Equation (12).

(12) $$h_{Cv}=0.38\times \frac{k}{d_{BG}}\times Re^{0.6}\times {Pr}^{1/3}$$

where k is the thermal conductivity of air (W m^−1^ K^−1^); $d_{BG}$ is the black globe diameter (m); Re is the Reynolds number (dimensionless); and Pr is the Prandtl number (dimensionless).

For the modeling of latent heat exchange ($E_{La}$, in W m^−2^), respiratory evaporation was considered as the sole mechanism of evaporative heat dissipation. Latent heat exchange was estimated using the empirical relationship proposed by Hellickson and Walker [35], as given by Equation (13), considering the climate chamber as an open thermodynamic system in which both mass and energy cross the system boundary.

(13) $$E_{La}=\rho \times v_{air}\times (W_{out}-W_{in})\times \lambda_{S}$$

where ρ is the air density (kg m^−3^); $v_{air}$ is the air velocity (m s^−1^); $W_{out}$ is the humidity ratio of the outlet air, estimated for the region near the exhaust outlet (kg water kg^−1^ dry air); $W_{in}$ is the humidity ratio of the inlet air, estimated for the region near the air conditioner outlet (kg water kg^−1^ dry air); and $\lambda_{S}$ is the latent heat of vaporization of water (J kg^−1^), assumed to be 2402 J kg^−1^ according to Bergman et al. [36].

The partial water vapor pressure of the inlet air ($p_{v,in}$, in kPa) and outlet air ($p_{v,out}$, in kPa) was estimated from the saturation vapor pressure and the relative humidity measured at each location, according to Equation (14):

(14) $$p_{v,in,out}=p_{Sat}\times \frac{RH}{100}$$

where $p_{Sat}$ is the saturation vapor pressure (kPa) and RH is the relative humidity (%).

The saturation vapor pressure ($p_{Sat}$, in kPa) was estimated using the empirical equation proposed by Tetens [37,38], as given by Equation (15).

(15) $$p_{Sat}=0.6108\times 10^{\left(\frac{7.50\times T_{A}}{237.30+T_{A}}\right)}$$

where $T_{A}$ is the air temperature (°C).

The humidity ratio of the inlet air ($W_{in}$, in kg water kg^−1^ dry air) and outlet air ($W_{out}$, in kg water kg^−1^ dry air) was subsequently calculated from the corresponding water vapor pressure as follows:

(16) $$W_{in,out}=0.622\times \left(\frac{p_{v,in,out}}{P-p_{v,in,out}}\right)$$

where P is the total atmospheric pressure (kPa).

The thermophysical properties used in the heat exchange modeling were estimated using equations expressed as a function of air temperature ($T_{A}$, in °C), as recommended by Silva [34]. The equations used to determine these properties are listed in Table 3.

### 2.4. Statistical Analysis

The experiment was conducted using a completely randomized design in a 4 × 3 factorial arrangement, consisting of four dietary inclusion levels of the macroalga *G. birdiae* (0.00, 3.00, 6.00, and 9.00%) and three air temperature conditions (25.00, 29.00, and 33.00 °C), resulting in 12 treatments. Each experimental unit consisted of one compartment of the cage battery housing 12 quails, with six replicates per treatment. Experimental data were initially organized, checked for consistency, and tabulated using Microsoft Excel^®^ (version 2606; Microsoft Corporation, Redmond, WA, USA) before being exported for statistical analysis. Prior to the inferential analyses, residual normality was assessed using the Shapiro–Wilk test. For the growing phase, week of evaluation was included as an additional fixed factor in the statistical model, together with dietary inclusion level and air temperature, including their two- and three-way interactions. When significant main effects or interactions were detected (*p* < 0.05), treatment means were compared using Tukey’s honestly significant difference test. Analysis of variance was performed using the GLM (General Linear Model) procedure in SAS OnDemand for Academics^®^ (version 9.4; SAS Institute Inc., Cary, NC, USA).

## 3. Results and Discussion

### 3.1. Heat Exchange Dynamics During the Growing Phase

During the growing phase, bird age (week of evaluation) significantly affected radiative heat exchange (E_Ra_), convective heat exchange (E_Cv_), total sensible heat exchange (E_SH_), respiratory latent heat exchange (E_La_), and total heat exchange (E_TH_) (Table 4), indicating progressive changes in heat exchange partitioning throughout bird development without altering the physiological function of thermoregulation. Between the first and fourth weeks, radiative heat exchange and respiratory latent heat exchange increased by 4.65% and 20.30%, respectively, whereas convective heat exchange and total sensible heat exchange decreased by 24.17% and 4.11%, respectively, resulting in an approximately 5.19% increase in total heat exchange (Table 4). The increase in E_TH_ indicates an enhanced overall capacity for heat dissipation throughout body development, accompanied by changes in the relative contribution of sensible and evaporative pathways. This response is consistent with the progressive development of thermoregulatory capacity reported for this stage of growth [10]. These findings further suggest that growth and physiological maturation promote a gradual reorganization of thermoregulatory mechanisms. Such behavior is expected because of the greater metabolic demands associated with body development, which require adjustments in the relative contribution of sensible and evaporative pathways to maintain homeothermy, as reported in recent studies on the ontogeny of thermoregulation in birds exposed to heat stress [11,12]. Within the sensible heat fraction, radiative heat exchange predominated over convective heat exchange throughout the growing phase, indicating that radiation was the primary pathway for sensible heat dissipation under the experimental conditions evaluated. This finding is consistent with the greater efficiency of radiative heat transfer in young birds exposed to favorable thermal gradients [19,39].

Air temperature (T_A_) also significantly affected heat exchange dynamics during the growing phase (Table 4). Increasing T_A_ from 25.00 to 33.00 °C reduced convective heat exchange (E_Cv_) by 69.20%, radiative heat exchange (E_Ra_) by 71.20%, and total sensible heat exchange (E_SH_) by 69.70%, while increasing respiratory latent heat exchange (E_La_) by 53.30%. As a result, total heat exchange (E_TH_) decreased by 38.20% (Table 4), demonstrating that increasing T_A_ progressively impairs the efficiency of sensible heat dissipation mechanisms. This response results from the reduction in the thermal gradient between the body surface and the surrounding environment, which simultaneously limits convective and radiative heat exchange while increasing the reliance on respiratory evaporation to maintain homeothermy [10,11]. This reorganization reflects a shift in the relative contribution of the different heat dissipation mechanisms rather than a change in the physiological function of thermoregulation, which remains directed toward maintaining body thermal balance [12,19].

However, although the increase in respiratory evaporative heat dissipation represents an important compensatory mechanism, it was insufficient to fully offset the reduction in sensible heat exchange, resulting in the lower overall heat dissipation capacity observed in the birds [11]. This response demonstrates that, even during the growing phase, increasing T_A_ promotes a progressive shift in the bioenergetic strategy of thermoregulation, characterized by the transition from the predominance of sensible heat dissipation to a greater contribution of evaporative heat loss.

In contrast to the effect of T_A_, dietary inclusion levels of *G. birdiae* did not significantly affect (*p* > 0.05) convective (E_Cv_), radiative (E_Ra_), sensible (E_SH_), latent (E_La_), or total heat exchange (E_TH_) during the growing phase (Table 4). Under the experimental conditions evaluated, dietary supplementation did not produce detectable changes in the bioenergetic partitioning of heat exchange or in the thermoregulatory variables assessed [26,40]. These findings indicate that the thermal responses observed during the growing phase were primarily determined by T_A_ rather than by dietary supplementation. Although no measurable effects of *G. birdiae* on heat exchange dynamics were detected, these results indicate only that dietary supplementation did not significantly modify the evaluated heat exchange variables under the experimental conditions of this study [22,41].

Collectively, the findings obtained during the growing phase demonstrate that heat exchange organization in Japanese quail is determined primarily by the interaction between physiological development and T_A_, whereas dietary inclusion of *G. birdiae* did not modify this response pattern (Table 4). As the birds grew and were exposed to higher T_A_, a progressive reorganization of heat dissipation pathways was observed, characterized by a reduced contribution of sensible heat exchange and an increased contribution of evaporative heat dissipation. This response reflects a physiological adaptation aimed at maintaining homeothermy despite the reduced thermal gradient between the bird and the surrounding environment [11,12]. The absence of detectable effects of dietary supplementation on heat exchange variables indicates that the thermoregulatory adjustments observed during the growing phase were primarily associated with increasing air temperature [21,22]. Within the conditions evaluated, *G. birdiae* supplementation did not measurably modify the bioenergetic organization of heat dissipation. These findings establish the conceptual basis for interpreting the laying phase, in which the onset of reproductive activity is accompanied by greater thermoregulatory demands and changes in the relative contribution of the different heat dissipation pathways [10,15].

### 3.2. Heat Exchange Dynamics During the Laying Phase

During the laying phase, T_A_ was the primary factor determining heat exchange dynamics in Japanese quail, significantly affecting E_Ra_, E_Cv_, E_SH_, E_La_, and E_TH_ (*p* < 0.0001), whereas supplementation with *G. birdiae* exerted a more limited effect, restricted to E_Ra_ (*p* = 0.0011), E_Cv_ (*p* = 0.0014), E_SH_ (*p* = 0.0011), and E_TH_ (*p* = 0.0013), with no significant interaction between the factors (*p* > 0.05; Table 5). Overall, the results obtained during the laying phase clearly indicate that air temperature was the primary factor governing the bioenergetic dynamics of heat exchange, whereas dietary supplementation with *Gracilaria birdiae* exerted comparatively limited effects and did not modify the overall thermoregulatory response. These findings quantitatively characterize the thermoregulatory adjustments observed under increasing T_A_, confirming patterns previously described for quails exposed to heat stress. In this context, the progressive reduction in sensible heat exchange, together with the simultaneous increase in evaporative heat dissipation, reflects a physiological reorganization of heat loss mechanisms aimed at maintaining thermal homeostasis. This response is consistent with the thermoregulatory adjustments previously described for quails exposed to heat stress [10,17,20].

The magnitude of these changes provides a quantitative characterization of the well-established shift in heat dissipation pathways across the thermal gradient evaluated (Table 5). Between 25.00 and 33.00 °C, E_SH_ decreased by 59.96%, from 55.47 to 22.21 W m^−2^, whereas E_La_ increased by 53.27%, from 23.73 to 36.37 W m^−2^ (Table 5). Consequently, E_TH_ decreased by 26.04% (Table 5), reflecting the reduced capacity of the quails to dissipate heat to the surrounding environment as air temperature approached body temperature [8,11]. Rather than indicating a previously unrecognized physiological response, these results quantitatively describe the functional redistribution of heat dissipation pathways already recognized in avian thermoregulation. This transition is consistent with the well-established physiological adaptation of birds exposed to heat stress and results from the progressive reduction in the thermal gradient between the body surface and the surrounding environment, which limits sensible heat dissipation and increases reliance on respiratory evaporation [10,42]. This response further confirms that, during the laying phase, a substantial proportion of metabolic energy is continuously allocated to maintenance processes and egg synthesis and formation, thereby reducing the physiological capacity of the quails to accommodate additional increases in heat production when exposed to hot environments. Consequently, even modest increases in environmental heat load shift the balance between heat production and heat dissipation more rapidly, making respiratory evaporation the primary compensatory mechanism for maintaining thermal homeostasis [8,43].

Among the components of sensible heat exchange, radiative heat exchange (E_Ra_) remained the primary pathway for heat dissipation throughout the laying phase (Table 5), accounting for approximately 76.49%, 77.02%, and 77.35% of E_SH_ at air temperatures of 25.00, 29.00, and 33.00 °C, respectively. Despite this predominance, E_Ra_ decreased by 59.51% between 25.00 and 33.00 °C, from 42.43 to 17.18 W m^−2^, demonstrating that radiative heat dissipation became progressively constrained as environmental heat load increased. This response indicates that, although radiation remained the predominant component of sensible heat exchange, its absolute contribution became insufficient to offset the increase in endogenous heat production during the laying phase, requiring a greater contribution of evaporative mechanisms to maintain thermal homeostasis [10,19,44]. This pattern is consistent with the thermoregulatory response described for birds exposed to heat stress, in which the reduction in radiative heat dissipation represents one of the earliest adaptations resulting from the progressive decline in the efficiency of sensible heat exchange under hot environmental conditions [14,42].

In contrast to the pronounced effects of T_A_, dietary inclusion of *G. birdiae* produced only modest changes in sensible heat exchange during the laying phase (Table 5). Radiative heat exchange (E_Ra_) decreased gradually from 31.32 W m^−2^ in the control diet to 28.92 W m^−2^ with 9.00% dietary inclusion, accompanied by reductions in E_Cv_, E_SH_, and E_TH_, whereas E_La_ remained unaffected (*p* = 0.0560). Although these reductions reached statistical significance, their magnitude remained biologically small when compared with the much larger changes induced by increasing T_A_. For example, dietary supplementation reduced E_Ra_, E_Cv_, E_SH_, and E_TH_ by approximately 7.00–8.00%, whereas increasing T_A_ from 25.00 to 33.00 °C reduced sensible heat exchange by nearly 60.00%. Therefore, these differences should be interpreted as subtle quantitative adjustments rather than biologically substantial modifications of the thermoregulatory strategy adopted by the birds under the experimental conditions evaluated. The absence of effects on respiratory evaporative heat loss further supports the interpretation that dietary supplementation did not modify the predominant thermoregulatory adjustments imposed by increasing T_A_, despite producing small quantitative changes in some components of sensible heat exchange [21,22,24]. These findings agree with previous studies indicating that marine macroalgae may support physiological responses under heat stress without substantially modifying the fundamental mechanisms of thermoregulation [26,45].

Collectively, these findings provide a quantitative bioenergetic characterization of the heat exchange adjustments associated with increasing T_A_, confirming patterns that are well established in avian thermoregulation while extending their quantitative description under the experimental conditions evaluated (Table 5). This response highlights the pronounced bioenergetic vulnerability of laying Japanese quail to heat stress and reinforces that nutritional interventions should be regarded as complementary, rather than alternative, strategies to the physiological mechanisms of thermoregulation [10,42,46]. Accordingly, the limited effects of dietary supplementation observed in the present study reinforce that improvements in the thermal environment remain the primary strategy for mitigating heat stress, whereas nutritional supplementation should be regarded as a complementary approach [15,22,24].

### 3.3. Physiological Responses Associated with Heat Exchange

The changes observed in heat exchange dynamics during the growing and laying phases (Table 4 and Table 5) were accompanied by physiological responses consistent with the progressive decline in the efficiency of sensible heat exchange and the increased recruitment of evaporative heat dissipation mechanisms. Overall, increasing T_A_ promoted an increase in respiratory rate (RR, breaths min^−1^), whereas body surface temperature (T_S_, °C) and cloacal temperature (T_C_, °C) exhibited more modest variations, consistent with the maintenance of homeothermy. This response was observed during both the growing and laying phases across the dietary inclusion levels of *G. birdiae*, although some significant effects and interactions involving dietary inclusion were detected for specific physiological variables (Table 6 and Table 7). Collectively, these findings reinforce that the physiological responses did not occur in isolation but rather reflected the thermoregulatory adjustments triggered by changes in the relative contribution of sensible and latent heat exchange previously described, thereby providing physiological support for the interpretation of the bioenergetic mechanisms discussed in this section.

RR was the physiological variable most sensitive to the transition between sensible and evaporative heat dissipation during both the growing and laying phases [12,14]. As increasing T_A_ reduced the thermal gradient between the body surface and the surrounding environment, thereby decreasing convective heat exchange (E_Cv_), radiative heat exchange (E_Ra_), and consequently total sensible heat exchange (E_SH_), the birds increased respiratory ventilation to enhance evaporative heat dissipation, as reflected by the increase in E_La_ described in the previous subsections [10,16,19]. This response was confirmed during the growing phase, in which increasing T_A_ from 25.00 to 33.00 °C increased RR by approximately 15.56%, from 81.34 to 93.99 breaths min^−1^ (*p* < 0.0001), whereas dietary inclusion of *G. birdiae* had no significant effect on this variable (Table 6). These findings demonstrate that RR was directly associated with the reorganization of heat dissipation pathways, enabling the birds to compensate for the pronounced reduction in sensible heat exchange (E_SH_) observed with increasing T_A_, which reached approximately 69.70% during the growing phase and 59.96% during the laying phase between 25.00 and 33.00 °C.

Increasing T_A_ significantly increased T_S_ during the growing phase, from 32.87 to 35.49 °C between 25.00 and 33.00 °C (*p* < 0.0001), with no effect of dietary inclusion levels of *G. birdiae* (Table 6). This response was directly associated with the reduction in the thermal gradient between the body surface and the surrounding environment, which decreased the efficiency of convective heat exchange (E_Cv_) and radiative heat exchange (E_Ra_), as demonstrated by the reduction in these heat dissipation pathways listed in Table 4 [11,47]. Under these conditions, the increase in T_S_ represents an expected peripheral response resulting from the lower efficiency of sensible heat transfer to the surrounding environment and the consequent accumulation of thermal energy at the body surface [12,26]. This adaptation promotes body heat redistribution and acts in concert with the increase in evaporative heat dissipation, contributing to delaying the rise in core body temperature even under greater thermal challenge [8,48]. Therefore, the integrated interpretation of Table 4 and Table 6 demonstrates that the increase in T_S_ does not, by itself, indicate impaired thermoregulatory capacity but rather represents a physiological consequence of the reorganization of heat dissipation pathways, whose effectiveness can be confirmed by evaluating T_C_.

In contrast to RR and T_S_ during the growing phase, T_C_ exhibited a smaller range of variation during both experimental phases (Table 6 and Table 7), indicating that the physiological adjustments triggered by increasing T_A_ were sufficient to maintain homeothermy [8,49]. This response indicates that the progressive increase in evaporative heat dissipation (E_La_), together with the reorganization of sensible heat exchange previously demonstrated by the reductions in E_Cv_, E_Ra_, and E_SH_ (Table 4 and Table 5), compensated for the limited capacity to dissipate heat through non-evaporative pathways, thereby restricting heat accumulation within the body core [10,11]. Therefore, the relative stability of T_C_ should not be interpreted as evidence of the absence of thermal challenge but rather as a consequence of the effectiveness of the thermoregulatory mechanisms recruited to maintain body temperature within the physiological range required for homeothermy [20,49]. Collectively, the findings listed in Table 4, Table 5, Table 6 and Table 7 demonstrate that the physiological changes observed represent adaptive responses to the bioenergetic reorganization of heat exchange, supporting the interpretation that enhanced evaporative heat dissipation constitutes the primary compensatory mechanism in response to the reduced efficiency of sensible heat exchange in Japanese quail.

The comparison between the growing and laying phases demonstrates that the greater intensity of the physiological responses observed during the laying phase resulted from the increased challenge imposed on body thermal balance [49,50]. Although the pattern of heat exchange reorganization was similar in both phases, laying birds exhibited a greater reliance on evaporative mechanisms to compensate for the reduced efficiency of sensible heat exchange (Table 4 and Table 5). This response is associated with increased body mass, greater metabolic heat production, and the high energy demand associated with egg formation, all of which reduce the physiological capacity for heat dissipation through sensible pathways [12,19,21]. Under these conditions, even small reductions in the efficiency of sensible heat exchange require greater recruitment of respiratory evaporation to maintain homeothermy, thereby explaining the more pronounced physiological responses observed during the laying phase [11,15]. Therefore, these findings demonstrate that physiological stage influences the magnitude of the thermoregulatory response without altering the predominant bioenergetic mechanism underlying adaptation to heat stress.

Collectively, these findings indicate that dietary supplementation with *G. birdiae* did not substantially modify the predominant physiological strategy of heat dissipation associated with increasing T_A_ [26]. This demonstrates that the thermoregulatory mechanisms remained preserved, indicating that dietary inclusion of up to 9.00% of the macroalga did not compromise either the maintenance of homeothermy or the reorganization of heat dissipation pathways under heat stress [15,24]. Thus, RR, T_S_, and T_C_ represent complementary physiological manifestations of the same adaptive process, in which the progressive reorganization of the relative contribution of sensible and evaporative heat exchange enables the birds to maintain homeothermy despite the reduced efficiency of heat dissipation through non-evaporative pathways.

### 3.4. Bioenergetic Integration and Study Implications

The integrated analysis of the results presented in Table 4, Table 5, Table 6 and Table 7 demonstrates that the response of Japanese quail to heat stress results from a coordinated reorganization of heat dissipation mechanisms, involving concurrent changes in heat exchange and physiological responses [11,13]. Collectively, the findings show that the progressive reduction in the contribution of sensible heat exchange was accompanied by greater recruitment of evaporative heat dissipation, whereas RR, T_S_, and T_C_ reflected the effectiveness of these adjustments in maintaining homeothermy. This reorganization differed between the growing and laying phases, demonstrating that physiological stage influences the bioenergetic distribution of heat exchange. In contrast, supplementation with *G. birdiae* preserved this functional organization without compromising the thermoregulatory mechanisms of the birds. Therefore, the integration of these findings advances the mechanistic understanding of heat stress adaptation in Japanese quail and establishes the conceptual basis for the biological and practical implications discussed in the following section.

The integration of the findings demonstrates that the redistribution of heat exchange observed throughout bird development represents not merely a response to increasing T_A_ but a reorganization of the relative contribution of radiative, convective, and evaporative heat dissipation pathways. As increasing T_A_ reduced the thermal gradient between the body surface and the surrounding environment, the efficiency of sensible heat exchange declined, leading to greater reliance on evaporative heat dissipation to maintain thermal balance. From this perspective, the association between heat exchange and physiological responses supports the interpretation of thermoregulation as an integrated bioenergetic process, in which RR, T_S_, and T_C_ represent complementary manifestations of the adjustments employed to maintain homeothermy [8,15]. This interpretation advances the understanding of the mechanisms underlying heat stress adaptation in Japanese quail and provides a physiological framework for the integrated evaluation of nutritional and environmental strategies aimed at mitigating its effects [9,51].

### 3.5. Limitations and Perspectives for Future Studies

The experimental design adopted in this study enabled strict environmental control throughout the experimental period, allowing the evaluation of thermoregulatory responses under well-defined thermal conditions. Nevertheless, because each air temperature was assigned to a single climate chamber, chamber-specific effects cannot be completely separated from the imposed thermal conditions. Although each cage compartment contained an independent group of quails and therefore constituted the observational unit for data collection for the physiological and heat exchange measurements, the thermal treatment itself was applied at the climate chamber level. Consequently, the biological observations should not be interpreted as fully independent experimental units for the temperature factor.

Another limitation of this study is that heat exchange was estimated using well-established biophysical models integrating environmental and physiological measurements rather than direct calorimetric determinations. Although these models provide a robust framework for quantifying convective, radiative, and evaporative heat fluxes under controlled environmental conditions, they rely on simplifying assumptions regarding heat transfer processes and therefore provide indirect estimates of thermal exchange. In addition, the present study did not include direct measurements of metabolic heat production, oxygen consumption, carbon dioxide production, or other metabolic indicators. Consequently, the physiological interpretation of heat exchange should be understood primarily from the integrated biophysical modeling approach adopted rather than from direct metabolic evidence. Future studies combining bioenergetic modeling with independent experimental validation and direct measurements of heat production would further strengthen model accuracy and support the interpretation of heat exchange dynamics under heat stress.

Although the controlled experimental design allowed the bioenergetic dynamics of heat exchange to be quantified under highly controlled conditions, the extrapolation of these findings to commercial production systems should be made with caution. In addition, the present findings are restricted to female Japanese quail (*Coturnix coturnix japonica*) evaluated under controlled climatic conditions and within the range of air temperatures investigated in this study. Therefore, caution should also be exercised when extrapolating these results to other poultry species, genetic backgrounds, physiological stages, or production environments that differ substantially from the experimental conditions adopted herein.

Under commercial conditions, factors such as air velocity, stocking density, microclimatic heterogeneity, and production level may modify the relative contribution of the different heat dissipation pathways [42,52]. Therefore, future studies should integrate bioenergetic modeling with productive, metabolic, and molecular responses to provide a more comprehensive understanding of the mechanisms underlying adaptation to heat stress. Photoperiod should also be considered in future investigations because it is recognized as an environmental factor capable of influencing feed intake, physiological responses, and thermoregulation. Evaluating different lighting programs under identical thermal conditions may help clarify their interaction with the bioenergetic dynamics of heat exchange. Such an approach may also clarify how metabolic responses contribute to the bioenergetic regulation of heat dissipation under heat stress. Furthermore, such studies may help clarify whether functional nutritional interventions influence thermoregulatory adaptation under different thermal environments and production conditions, thereby providing a stronger experimental basis for future investigations of thermoregulatory adaptation under diverse production conditions.

## 4. Conclusions

Heat stress markedly altered the bioenergetic dynamics of heat exchange in Japanese quail by progressively reducing sensible heat dissipation and increasing reliance on respiratory evaporative heat loss while maintaining body temperature within the physiological range required for homeothermy. These findings demonstrate the central role of air temperature in regulating thermoregulatory heat exchange under controlled environmental conditions.

Dietary inclusion of up to 9.00% *Gracilaria birdiae* exerted only limited effects on the quantified heat exchange pathways and did not modify the predominant thermoregulatory adjustments associated with increasing air temperature. These findings indicate that supplementation did not impair the physiological mechanisms involved in heat dissipation under the conditions evaluated, although no direct evidence of additional thermophysiological benefits was demonstrated.

Collectively, these findings provide a quantitative characterization of the bioenergetic redistribution of heat exchange in Japanese quail under controlled thermal conditions, contributing to the understanding of the integration between physiological and biophysical responses during heat stress. They also provide a mechanistic framework for future studies investigating thermoregulatory responses and for evaluating complementary nutritional strategies in Japanese quail under a broader range of experimental and commercial conditions. Their interpretation should, however, be limited to the controlled experimental conditions adopted in the present study until these findings have been validated under commercial production systems, different genetic backgrounds, and a wider range of environmental conditions.

## Figures and Tables

**Table 1:** Ingredient composition (%) and calculated nutritional composition of the experimental diets containing different dietary inclusion levels of *Gracilaria birdiae* for Japanese quail (*Coturnix coturnix japonica*) during the experimental period.

Ingredient (%)	Dietary Inclusion Level of *Gracilaria birdiae* (%)			
	0.00	3.00	6.00	9.00
Corn	58.37	54.34	50.30	46.28
Soybean meal	34.16	34.13	34.16	34.21
*Gracilaria birdiae*	0.00	3.00	6.00	9.00
Dicalcium phosphate	0.14	0.14	0.14	0.14
Soybean oil	2.33	3.89	4.40	5.37
Vitamin-mineral premix ^1^	5.00	5.00	5.00	5.00
**Total**	**100.00**	**100.00**	**100.00**	**100.00**
**Calculated nutritional composition**				
Metabolizable energy (kcal kg^−1^)	3050.00	3050.00	3050.00	3050.00
Crude protein (%)	22.00	22.00	22.00	22.00
Total calcium (%)	1.04	1.19	1.35	1.51
Available phosphorus (%)	0.34	0.34	0.34	0.34
Sodium (%)	0.219	0.226	0.234	0.242
Arginine (%)	1.30	1.29	1.27	1.25
Threonine (%)	0.70	0.69	0.68	0.67
Isoleucine (%)	0.81	0.80	0.79	0.78
Tryptophan (%)	0.24	0.24	0.23	0.23
Valine (%)	0.89	0.87	0.86	0.84
Leucine (%)	1.66	1.62	1.58	1.54
Lysine (%)	1.14	1.14	1.13	1.12
Methionine (%)	0.34	0.34	0.33	0.32
Methionine + cystine (%)	0.64	0.63	0.62	0.60

^1^ Values represent the calculated composition of the experimental diets formulated to meet the nutritional requirements of Japanese quail during the growing and laying phases, according to Rostagno et al. [28].

**Table 2:** Mineral composition, bioactive compound profile, and proximate composition of the marine macroalga *Gracilaria birdiae* used in the experimental diets for Japanese quail (*Coturnix coturnix japonica*).

Minerals (mg 100 g^−1^)				BC (mg 100 g^−1^)		Proximate Composition (%)	
Na	128.14	Ca	4211.39	TPC	170.16	CP	14.47
Mg	335.43	Mn	3.85	TF	44.46	MC	3.47
Al	207.01	Fe	126.06	TAn	14.21	TL	5.49
Si	519.55	Cu	0.47	TChl	1.98	ASH	45.80
K	38.00	Zn	0.10	TCar	51.54	TCa	49.30

Values are presented as the mean of three independent analytical determinations. The analyses were performed at the Laboratory of Storage and Processing of Agricultural Products (LAPPA/UAEA/UFCG) and the Food Engineering Laboratory (LEA/UFCG). ASH—total ash; BC—bioactive compounds; CP—crude protein; MC—moisture; TAn—total anthocyanins; TCa—total carbohydrates; TCar—total carotenoids; TChl—total chlorophylls; TF—total flavonoids; TL—total lipids; TPC—total phenolic compounds.

**Table 3:** Equations and input variables used to determine the thermophysical properties of air employed in the heat exchange modeling between Japanese quail and the surrounding environment.

Thermophysical Property	Equation	Unit
Specific heat capacity of air	$c_{p}=1005.524+0.033714\times T_{A}$	J kg^−1^ K^−1^
Thermal conductivity of air	$k=0.024324+6.2909\times 10^{-5}\times T_{A}$	W m^−1^ K^−1^
Air density	$\rho =1.289764-0.004111\times T_{A}$	kg m^−3^
Kinematic viscosity of air	$\nu =1.32909\times 10^{-5}+9\times 10^{-8}\times T_{A}$	m^2^ s^−1^

$T_{A}$—air temperature (°C).

**Table 4:** Analysis of variance results and mean values for radiative heat exchange (E_Ra_), convective heat exchange (E_Cv_), total sensible heat exchange (E_SH_), respiratory latent heat exchange (E_La_), and total heat exchange (E_TH_) in Japanese quail (*Coturnix coturnix japonica*) during the growing phase (weeks 1–4; 15–42 days of age) subjected to different air temperatures (T_A_, °C) and dietary inclusion levels of *Gracilaria birdiae*.

Effect	Heat Exchange Components (W m^−2^)				
	E_Ra_	E_Cv_	E_SH_	E_La_	E_TH_
**Week of evaluation—W**					
1st	26.87 b	11.79 a	38.66 a	23.43 d	62.09 b
2nd	27.26 ab	10.11 b	37.37 ab	24.36 c	61.99 b
3rd	26.66 b	9.08 c	35.73 c	27.48 b	63.21 b
4th	28.12 a	8.94 c	37.07 bc	28.24 a	65.31 a
**Dietary inclusion level of the macroalga *Gracilaria birdiae*** **—L**					
0.00	27.27	9.95	37.22	25.92	63.14
3.00	27.63	10.14	37.77	25.84	63.61
6.00	27.09	9.94	37.03	25.95	62.98
9.00	26.91	9.91	36.82	26.07	62.89
**Air temperature—T_A_**					
25.00	43.43 a	16.27 a	59.70 a	20.60 c	80.30 a
29.00	24.86 b	8.99 b	33.85 b	25.66 b	59.51 b
33.00	13.38 c	4.68 c	18.06 c	31.57 a	49.63 c
SEM	2.22	0.93	3.15	0.57	2.81
***p*-value**					
W	0.0005	<0.0001	<0.0001	<0.0001	<0.0001
L	0.2549	0.4256	0.3087	0.1058	0.4285
T_A_	<0.0001	<0.0001	<0.0001	<0.0001	<0.0001
W × L	0.9423	0.926	0.9407	0.7185	0.9305
W × T_A_	<0.0001	<0.0001	<0.0001	<0.0001	<0.0001
L × T_A_	0.3357	0.553	0.392	0.9307	0.2382
W × L × T_A_	0.4879	0.4963	0.486	0.2727	0.5232

Means followed by different lowercase letters within the same column and factor differ significantly according to Tukey’s test (*p* < 0.05). *p*-values refer to the main effects and their respective interactions evaluated by the analysis of variance. SEM—standard error of the mean.

**Table 5:** Analysis of variance results and mean values for radiative heat exchange (E_Ra_), convective heat exchange (E_Cv_), total sensible heat exchange (E_SH_), respiratory latent heat exchange (E_La_), and total heat exchange (E_TH_) in Japanese quail (*Coturnix coturnix japonica*) during the laying phase (49–112 days of age) subjected to different air temperatures (T_A_, °C) and dietary inclusion levels of *Gracilaria birdiae*.

Effect	Heat Exchange Components (W m^−2^)				
	E_Ra_	E_Cv_	E_SH_	E_La_	E_TH_
**Dietary inclusion level of the macroalga *Gracilaria birdiae*—L**					
0.00	31.32 a	9.43 a	40.75 a	29.89 ab	70.64 a
3.00	30.81 ab	9.31 a	40.12 ab	28.78 b	69.91 ab
6.00	29.53 bc	8.92 ab	38.44 bc	29.99 a	68.44 bc
9.00	28.92 c	8.70 b	37.62 c	29.93 ab	67.54 c
**Air temperature—T_A_**					
25.00	42.43 a	13.04 a	55.47 a	23.73 c	79.20 a
29.00	30.82 b	9.20 b	40.02 b	29.59 b	69.61 b
33.00	17.18 c	5.03 c	22.21 c	36.37 a	58.58 c
SEM	1.91	0.59	2.50	0.23	2.46
***p*-value**					
L	0.0011	0.0014	0.0011	0.0560	0.0013
T_A_	<0.0001	<0.0001	<0.0001	<0.0001	<0.0001
L × T_A_	0.9948	0.9886	0.9953	0.8124	0.9923

Means followed by different lowercase letters within the same column and factor differ significantly according to Tukey’s test (*p* < 0.05). SEM—standard error of the mean.

**Table 6:** Physiological responses associated with bioenergetic heat exchange dynamics in Japanese quails during the growing phase (15–42 days of age) subjected to different air temperatures and dietary inclusion levels of *Gracilaria birdiae*.

Effect	Physiological Variable		
	RR (breaths min^−1^)	T_S_ (°C)	T_C_ (°C)
**Dietary inclusion level of the macroalga *Gracilaria birdiae*—L**			
0.00	88.90	34.17	41.52
3.00	89.13	34.12	41.52
6.00	88.98	34.12	41.27
9.00	87.85	34.08	41.27
**Air temperature—T_A_**			
25.00	81.34 c	32.87 c	41.24 b
29.00	90.82 b	34.01 b	41.28 b
33.00	93.99 a	35.49 a	41.67 a
SEM	3.46	0.29	0.26
***p*-value**			
L	0.673	0.780	0.002
T_A_	<0.0001	<0.0001	<0.0001
L × T_A_	0.366	0.093	0.003

Means followed by different lowercase letters within the same column and factor differ significantly according to Tukey’s test (*p* < 0.05). RR—respiratory rate (breaths min^−1^); T_S_—body surface temperature (°C); T_C_—cloacal temperature (°C); SEM—standard error of the mean.

**Table 7:** Physiological responses associated with bioenergetic heat exchange dynamics in Japanese quail during the laying phase (49–112 days of age) subjected to different air temperatures and dietary inclusion levels of *Gracilaria birdiae*.

Effect	Physiological Variable		
	RR (breaths min^−1^)	T_S_ (°C)	T_C_ (°C)
**Dietary inclusion level of the macroalga *Gracilaria birdiae*—L**			
0.00	87.34	34.29 a	41.35
3.00	87.64	34.19 ab	41.35
6.00	88.07	33.99 bc	41.33
9.00	88.73	33.83 c	41.35
**Air temperature—T_A_**			
25.00	84.17 c	32.38 c	41.01 c
29.00	87.58 b	34.21 b	41.43 b
33.00	92.07 a	35.64 a	41.59 a
SEM	1.86	0.31	0.16
***p*-value**			
L	0.1413	0.0002	0.9616
T_A_	<0.0001	<0.0001	<0.0001
L × T_A_	0.0062	0.7229	0.0125

Means followed by different lowercase letters within the same column and factor differ significantly according to Tukey’s test (*p* < 0.05). RR—respiratory rate (breaths min^−1^); T_S_—body surface temperature (°C); T_C_—cloacal temperature (°C); SEM—standard error of the mean.

## Data Availability

The datasets supporting the findings of this study are available from the corresponding author upon reasonable request.

## Abbreviations

The following abbreviations are used in this manuscript:

ASH	Total ash (%)
BC	bioactive compounds (mg 100 g^−1^)
CP	Crude protein (%)
c_p_	Specific heat capacity of air (J kg^−1^ K^−1^)
d_B_	Mean body diameter (m)
d_BG_	Black globe diameter (m)
EPS	Expanded polystyrene
E_Cv_	Convective heat exchange (W m^−2^)
E_La_	Respiratory latent heat exchange (W m^−2^)
E_Ra_	Radiative heat exchange (W m^−2^)
E_SH_	Total sensible heat exchange (W m^−2^)
E_TH_	Total heat exchange (W m^−2^)
k	Thermal conductivity of air (W m^−1^ K^−1^)
m	Mean body weight (g)
MC	Moisture (%)
Nu	Nusselt number (dimensionless)
CEUA	Animal Ethics Committee
h_Cv_	Globe convective heat transfer coefficient (W m^−2^ K^−1^)
L	Dietary inclusion level of the macroalga *Gracilaria birdiae*
LaCRA	Laboratory of Rural Buildings and Environment
LAPPA	Laboratory of Storage and Processing of Agricultural Products
LEA	Food Engineering Laboratory
Pr	Prandtl number (dimensionless)
p_Sat_	Saturation vapor pressure (kPa)
p_v,in_	Partial water vapor pressure of the inlet air (kPa)
p_v,out_	Partial water vapor pressure of the outlet air (kPa)
Re	Reynolds number (dimensionless)
r_Cv_	Boundary layer resistance to convective heat transfer (m^2^ K W^−1^)
RH	Relative humidity (%)
RR	Respiratory rate (breaths min^−1^)
r_Ra_	Boundary layer resistance to radiative heat transfer (m^2^ K W^−1^)
SEM	Standard error of the mean
TAn	Total anthocyanins (mg 100 g^−1^)
T_A_	Air temperature (°C or K, as specified)
T_back_	Back surface temperature (°C or K)
T_BG_	Black globe temperature (K)
TCa	Total carbohydrates (%)
T_C_	Cloacal temperature (°C)
TCar	Total carotenoids (mg 100 g^−1^)
TChl	Total chlorophylls (mg 100 g^−1^)
TF	Total flavonoids (mg 100 g^−1^)
T_head_	Head surface temperature (°C)
TL	Total lipids (%)
T_MR_	Mean radiant temperature (K)
TPC	Total phenolic compounds (mg 100 g^−1^)
T_S_	Body surface temperature (°C or K)
T_shank_	Shank surface temperature (°C)
T_wing_	Wing surface temperature (°C)
$\bar{T_{M}}$	Mean of T_S_ and T_MR_ (K)
UAEA	Academic Unit of Agricultural Engineering
UFCG	Federal University of Campina Grande
v_air_	Air velocity (m s^−1^)
W	Week of evaluation
W_out_	Humidity ratio of the outlet air (kg water kg^−1^ dry air)
W_in_	Humidity ratio of the inlet air (kg water kg^−1^ dry air)
$\varepsilon_{S}$	Surface emissivity of the feathers
λ_S_	Latent heat of vaporization of water (J kg^−1^)
ν	Kinematic viscosity of air (m^2^ s^−1^)
ρ	Air density (kg m^−3^)
σ	Stefan–Boltzmann constant (5.67051 × 10^−8^ W m^−2^ K^−4^)
